# T Helper and Cytotoxic T Cells Play an Important Role in Acute Gastric Injury

**DOI:** 10.3390/diseases13110374

**Published:** 2025-11-15

**Authors:** Irfan F. Corovic, Jelena M. Pantic, Isidora A. Stanisavljevic, Sladjana M. Pavlovic, Nemanja U. Jovicic, Ivan P. Jovanovic, Gordana D. Radosavljevic, Bojana J. Simovic Markovic

**Affiliations:** 1 Center for Molecular Medicine and Stem Cell Research, Faculty of Medical Sciences, University of Kragujevac, 34000 Kragujevac, Serbia; ira.corovic@gmail.com (I.F.C.); panticjelena55@gmail.com (J.M.P.); isidorastanisavljevic97@gmail.com (I.A.S.); sladjadile@gmail.com (S.M.P.); ivanjovanovic77@gmail.com (I.P.J.); perun.gr@gmail.com (G.D.R.); 2 Department of Internal Medicine, General Hospital of Novi Pazar, 36300 Novi Pazar, Serbia; 3 Department of Histology and Embryology, Faculty of Medical Sciences, University of Kragujevac, 34000 Kragujevac, Serbia; nemanjajovicic.kg@gmail.com; 4 Faculty of Medicine, University of East Sarajevo, 73300 Foca, Bosnia and Herzegovina

**Keywords:** peptic ulcer, acute gastric injury, CD4^+^ lymphocytes, CD8^+^ lymphocytes, T helper lymphocytes, cytotoxic lymphocytes

## Abstract

Background: Inflammation plays a central role in the formation of peptic ulcers, yet the contribution of cellular immunity remains poorly defined. This study aimed to clarify the contribution of cellular immunity to acute gastric mucosal injury. Methods: BALB/c mice received 80% ethanol via oral gavage to induce acute gastric injury. Stomachs were examined macroscopically and histologically, and gastric tissues were analyzed by qPCR, ELISA, and flow cytometry for cytokine expression, immune cell infiltration, and apoptosis. Results: Administration of ethanol exacerbated acute gastric injury in mice, as evidenced by extensive macroscopic lesions and severe disruption of mucosal architecture. This damage was accompanied by marked infiltration of CD11c^+^ dendritic cells, together with an increased frequency of CD86-expressing and IL-12-producing dendritic cells. In addition, there was greater accumulation of both CD4^+^ and CD8^+^ T lymphocytes, including elevated numbers of CD4^+^ and CD8^+^ cells producing IFN-γ and IL-17, as well as CD8^+^CD107a^+^ cytotoxic cells. Alongside these cellular alterations, ethanol exposure was accompanied by elevated levels of pro-inflammatory cytokines (IL-1β, TNF-α, IL-17, and IFN-γ) in gastric tissue. In parallel, ethanol exposure also promoted epithelial cell apoptosis, further contributing to mucosal deterioration. Conclusions: Our findings reveal for the first time that both CD4^+^ and CD8^+^ T cells participate in sterile ethanol-induced acute gastric injury, emphasizing cellular immunity as an important yet insufficiently studied contributor to mucosal damage and highlighting the necessity for further mechanistic and translational research.

## 1. Introduction

Peptic ulcer disease is characterized as a defect in the stomach or duodenal mucosa that spreads to the submucosa and remains a highly prevalent condition worldwide, affecting nearly 10% of the population. Despite advances in diagnosis and therapy, it continues to represent a significant public health and financial strain [1]. This condition arises due to an imbalance between harmful luminal factors, such as hydrochloric acid and pepsin, and the protective functions of the mucosal barrier. The well-recognized causes include infection by *Helicobacter pylori*, the use of non-steroidal anti-inflammatory drugs (NSAIDs), and the consumption of alcohol and tobacco, alongside psychological stress [2]. Innate immunity, with neutrophils as the predominant effector cells, plays a decisive role in driving tissue injury during ulcer formation. These innate responses not only initiate but also amplify the inflammatory cascade, thereby establishing inflammation as a key pathogenic mechanism underlying gastric mucosal damage [3,4]. Peptic ulcer disease is characterized by epigastric pain accompanied by dyspeptic features, including postprandial fullness, early satiety, bloating, and nausea, whereas the development of complications such as perforation, gastrointestinal bleeding, or obstruction markedly worsens clinical outcomes. Management primarily relies on proton pump inhibitor-based acid suppression and *H. pylori* eradication regimens [5]. However, increasing attention is being directed toward strategies that enhance epithelial barrier integrity and modulate mucosal immune mechanisms [6,7].

Adaptive cellular immunity, mediated by T lymphocytes, is fundamental to gastrointestinal homeostasis, calibrating tolerance to the commensal microbiota while maintaining robust defense against pathogens [8]. Disruption of these regulatory networks compromises epithelial barrier integrity and triggers self-perpetuating cycles of mucosal injury and inflammation. Cellular immunity is involved in the pathogenesis of numerous gastrointestinal diseases through context-dependent T-cell differentiation and effector responses. In inflammatory bowel disease, for example, a dynamic interplay among Th1, Th2, Th17, and cytotoxic T-cell populations, combined with insufficient regulatory control, drives the transition from acute inflammation to chronic tissue remodeling and fibrosis [9]. Conversely, the contribution of cellular immunity in gastric pathology has been predominantly explored in chronic gastric conditions, including *H. pylori* infection [10,11], autoimmune gastritis, and gastric cancer [12,13]. These studies have highlighted the roles of T helper subsets and cytotoxic T lymphocytes in long-term mucosal remodeling, persistent inflammation, and malignant transformation. However, whether acquired immune cells, specifically CD4^+^ and CD8^+^ T lymphocytes, participate in the pathogenesis of acute gastric injury remains poorly understood.

Therefore, the aim of the present study was to investigate the role of cellular adaptive immunity in a murine model of ethanol-induced acute gastric mucosal damage.

## 2. Materials and Methods

### 2.1. Animals

Six to eight-week-old BALB/c mice were utilized for the study. The mice were kept in the animal facilities at the Faculty of Medical Sciences, University of Kragujevac, Serbia. All experimental procedures were conducted in accordance with the guidelines approved by the Animal Ethics Committee at the Faculty of Medical Sciences, University of Kragujevac, Serbia. The mice were housed in an environment with controlled temperature and a 12 h light-dark cycle, and they were given access to standard laboratory food *ad libitum*.

### 2.2. Induction of Gastric Injury

Acute gastric injury was induced in BALB/c mice by intragastric gavage of 80% ethanol (10 mL/kg). Control mice received phosphate-buffered saline (PBS) (Thermo Fisher Scientific, Waltham, MA, USA) at an equivalent volume. Three hours post-gavage, mice were sacrificed by cervical dislocation. Animals were fasted for 24 h with free access to water prior to ethanol and PBS administration [14,15].

### 2.3. Evaluation of Gastric Mucosal Damage

For both macroscopic and histological assessments, the stomachs were removed from euthanized mice, cut along the greater curvature, and washed with PBS. The dimensions of the lesions were evaluated by photographing the inner stomach surface and analyzing the images with the ImageJ software program (version 1.53e, National Institute of Health, Bethesda, MD, USA). The gastric lesion score (%) was determined using the formula: gastric lesion score (%) = [area of damaged mucosa (mm^2^)/total mucosal area (mm^2^)] × 100 [16]. For histological evaluation, the isolated stomach tissues were fixed in 4% paraformaldehyde and subsequently dehydrated through a graded alcohol series. After embedding in paraffin, the tissues were sectioned into 4–5 µm thick slices using a microtome Leica RM 2135 (Leica Biosystems, Nussloch, Wetzlar, Germany) and stained with hematoxylin and eosin (H&E). Histopathological assessment was performed by scoring epithelial cell destruction (0–3), mucosal edema (0–3), hemorrhagic injury (0–3), and infiltration of inflammatory cells (0–3). Scores from these parameters were combined to produce a cumulative histological activity index (HAI), with a maximum achievable value of 12, as previously described [17].

### 2.4. Measurement of Cytokines in Tissue Homogenates

Commercial ELISA kits (R&D Systems, Minneapolis, MN, USA) were utilized to assess the levels of TNF-α, IL-1β, IL-17, IFN-γ, and IL-10 in the gastric tissue homogenates of both control and ethanol-treated animals. The preparation of gastric homogenates was done following previously established methods [18].

### 2.5. RNA Extraction and Real-Time PCR Assessment

Total RNA was isolated from gastric tissue samples with TRIzol reagent (Invitrogen, Carlsbad, CA, USA) following the manufacturer’s instructions. One microgram of RNA was used for reverse transcription into complementary DNA (cDNA) employing the High-Capacity cDNA Reverse Transcription Kit (Applied Biosystems, Foster City, CA, USA). Quantitative real-time PCR (qRT-PCR) was performed using Power SYBR Green Master Mix (Applied Biosystems, Foster City, CA, USA) together with gene-specific primers for Bax (forward 5′-ACACCTGAGCTGACCTTG-3′, reverse 5′-AGCCCATGATGGTTCTGATC-3′), caspase-3 (forward 5′-AAATTCAAGGGACGGGTCAT-3′, reverse 5′ ATTGACACAATACACGGGATCTGT-3′), and β-actin (forward 5′-AGCTGCGTTTTACACCCTTT-3′, reverse 5′-AAGCCATGCCAATGTTGTCT-3′), which served as an internal control. All primers were custom-synthesized and quality-controlled by Metabion GmbH (Planegg, Germany) and Invitrogen (Carlsbad, CA, USA). The thermal cycling protocol consisted of an initial denaturation at 95 °C for 10 min, followed by 40 cycles of denaturation at 95 °C for 15 s and annealing/extension at 60 °C for 60 s, performed on a Mastercycler ep realplex instrument (Eppendorf, Hamburg, Germany). Relative mRNA expression levels were calculated using the 2^−(Ct−Ctactin)^ method, where Ct corresponds to the cycle threshold of the target gene and Ctactin refers to the threshold cycle of the reference gene [19].

### 2.6. Isolation and Flow Cytometric Analysis of Gastric Tissue–Infiltrating Immune Cells

Immune cells were isolated from the gastric lamina propria using a protocol adapted from previously published methods [20]. Gastric tissues were excised, cut open along the longitudinal axis, and extensively flushed with RPMI-1640 medium (Thermo Fisher Scientific, Waltham, MA, USA) supplemented with L-glutamine (Sigma-Aldrich, Darmstadt, Germany) and 5% fetal bovine serum (FBS) (PAN-Biotech, Aidenbach, Germany) to clear luminal residues. To facilitate epithelial cell removal, the samples were incubated for 30 min in calcium- and magnesium-free Hank’s balanced salt solution (HBSS) (Sigma-Aldrich, Darmstadt, Germany) containing 1 mM EDTA (Sigma-Aldrich, Darmstadt, Germany), 1 mM dithiothreitol (DTT) (Thermo Fisher Scientific, Waltham, MA, USA), and 2% FBS. Subsequently, the tissue was mechanically fragmented and digested for an additional 30 min with an enzyme cocktail consisting of collagenase IV (0.5 mg/mL) (Thermo Fisher Scientific, Waltham, MA, USA) and DNase I (200 μg/mL) (Roche Diagnostics GmbH, Mannheim, Germany) at 37 °C under continuous agitation (250 rpm). The resulting suspensions were filtered through 40 μm nylon cell strainers, washed twice, and finally resuspended in RPMI-1640 supplemented with 5% FBS. A total of 1 × 10^6^ cells from each sample were prepared for flow cytometric analysis. Cells were stained with fluorochrome-conjugated monoclonal antibodies against CD11c, CD4, CD8, and CD86 (FITC, PE, PerCP, or APC; BD Biosciences, San Jose, CA, USA), following the supplier’s recommendations. For intracellular staining, suspensions were stimulated at 37 °C for 4 h with phorbol 12-myristate 13-acetate (50 ng/mL; Sigma-Aldrich), ionomycin (500 ng/mL; Sigma-Aldrich), and GolgiStop (1 μg/mL; BD Pharmingen, Franklin Lakes, NJ, USA). After surface labeling, cells were fixed, permeabilized, and incubated with antibodies specific for IL-12, IFN-γ, IL-17, and CD107a (FITC, PE, PerCP, APC; BD Biosciences) using a fixation/permeabilization kit. Data acquisition was performed on a FACSCalibur flow cytometer (BD Biosciences), and analyses were conducted with FlowJo software (version 10.7.2, Tree Star Inc., Ashland, OR, USA).

### 2.7. Statistical Analysis

Data are presented as mean ± standard error of the mean (SEM). Prior to statistical comparison, data distribution was assessed using the Shapiro–Wilk test. For normally distributed data, comparisons were performed using Student’s *t*-test. For data that did not meet normality criteria, non-parametric tests were applied, including the Mann–Whitney U test for two-group comparisons. Statistical analyses were performed using SPSS 26.0 for Windows (SPSS Inc., Chicago, IL, USA). A *p*-value < 0.05 was considered statistically significant.

## 3. Results

### 3.1. Ethanol Exacerbates Gastric Mucosal Injury Through Inflammation and Apoptosis

**Figure 1:**
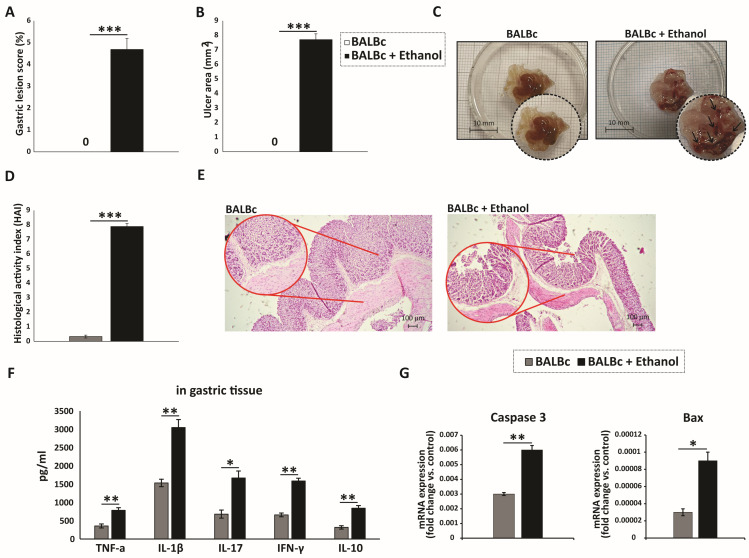
Ethanol-induced gastric mucosal injury marked by pronounced inflammation and epithelial apoptosis: (**A**) Macroscopic images of gastric mucosa showing the extent of injury after ethanol exposure. (**B**,**C**) Quantitative analysis of macroscopic damage: (**B**) gastric lesion score (%) and (**C**) ulcer area (mm^2^) measured from digital images using ImageJ. (**D**) HAI scores of tissue injury. (**E**) H&E-stained sections illustrating epithelial integrity, inflammatory infiltration, and gastric tissue architecture across groups. (**F**) Gastric tissue levels of IL-1β, TNF-α, IL-17, IFN-γ, and IL-10. (**G**) qRT-PCR analysis of Bax and caspase-3 mRNA expression in gastric tissue. Data are expressed as mean ± SEM; *n* = 10 mice per group. * *p* < 0.05, ** *p* < 0.01, *** *p* < 0.001. For score data (**A**,**D**), statistical analysis was performed using the non-parametric Mann–Whitney U test.

All ethanol-treated mice developed severe acute gastric injury with comparable macroscopic changes, indicating gross epithelial damage. Macroscopic evaluation was performed to assess the severity of gastric injury in the gastric mucosa of stomach specimens (Figure 1A–C). Intragastric administration of 80% ethanol induced severe hemorrhagic and ulcerated lesions in mice, implicating profound gastric damage following ethanol exposure. In contrast, the gastric mucosa of the control group appeared intact and light red, with normal morphology. Histological evaluation demonstrated that ethanol-treated mice developed pronounced gastric mucosal injury with marked epithelial cell loss and detachment, while the glandular layer appeared distorted and fragmented. Prominent hemorrhagic lesions were evident, together with mucosal edema and congestion of blood vessels. Inflammatory infiltrates were widely distributed throughout the damaged mucosa and extended into the submucosa (Figure 1D,E). In contrast, the control group displayed an almost intact mucosal layer, with preserved epithelial lining and typical gastric histoarchitecture, including well-organized glands. Moreover, ethanol administration markedly increased the levels of pro-inflammatory cytokines (TNF-α, IL-1β, IL-17, and IFN-γ), along with the anti-inflammatory cytokine IL-10, in the gastric tissue of WT mice compared with controls (Figure 1F).

It is well established that ethanol exerts a direct cytotoxic effect on gastric epithelial cells, primarily by inducing apoptosis [21]. The Bcl-2 family proteins, particularly the anti-apoptotic Bcl-2 and the pro-apoptotic Bax, are key regulators of the apoptotic pathway [22]. In our study, the expression level of the pro-apoptotic protein Bax in gastric mucosal tissue was significantly elevated in ethanol-treated mice compared with the control group (Figure 1G). Furthermore, caspase-3, a central executioner of apoptosis [22], displayed markedly increased activity in the ethanol-treated group relative to controls (Figure 1G). Collectively, these findings indicate that ethanol administration triggers pronounced gastric epithelial cell death via apoptosis, as evidenced by the upregulation of pro-apoptotic mediators including Bax and caspase-3.

### 3.2. Ethanol Induces an Accumulation and Functional Activation of Dendritic Cells (DCs)

**Figure 2:**
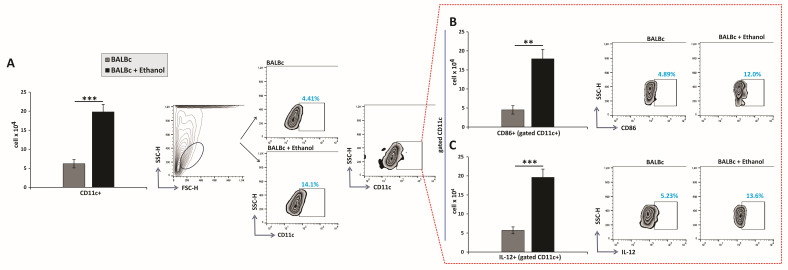
Ethanol triggers recruitment and activation of dendritic cells in the gastric mucosa: (**A**–**C**) Total numbers, subsets, and representative flow cytometry dot plots of DCs. (**A**) Total DC count. Quantification of DC subsets expressing (**B**) CD86 and (**C**) secreting IL-12. Mean values ± SEM are shown; *n* = 10 mice per group. * *p* < 0.05, ** *p* < 0.01, *** *p* < 0.001.

The total number of CD11c^+^ cells was significantly increased in the gastric lamina propria of ethanol-treated mice compared with controls (Figure 2A). Concomitantly, CD11c^+^ DCs from ethanol-exposed animals exhibited markedly increased expression of the costimulatory molecule CD86, indicating an enhanced antigen-presenting capacity (Figure 2B). Moreover, ethanol treatment significantly augmented the production of the pro-inflammatory cytokine IL-12 within the CD11c^+^ population (Figure 2C). These findings demonstrate that ethanol not only expands the pool of gastric DCs but also potentiates their immunostimulatory function, thereby contributing to the amplification of mucosal inflammation.

### 3.3. Ethanol Administration Enhances the Infiltration and Activation of T Lymphocytes

**Figure 3:**
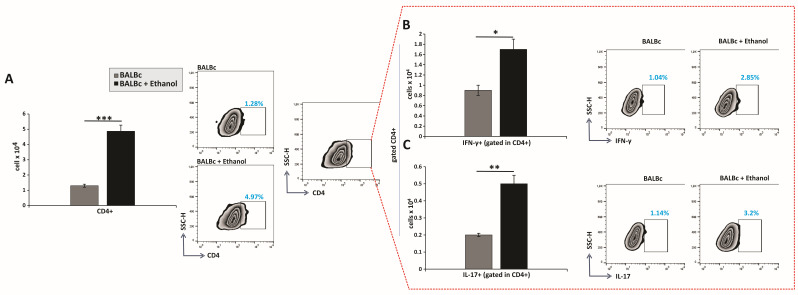
Ethanol promotes the pro-inflammatory capacity of CD4^+^ T lymphocytes: (**A**–**C**) Total numbers, subsets, and representative flow cytometry dot plots of CD4^+^ T cells. (**A**) Total CD4^+^ T-cell count. Quantification of CD4^+^ subsets producing (**B**) IFN-γ and (**C**) IL-17. Results are displayed as mean ± SEM; *n* = 10 mice per group. * *p* < 0.05, ** *p* < 0.01, *** *p* < 0.001.

The total number of CD4^+^ T cells in the gastric lamina propria was significantly increased in ethanol-treated mice compared with control animals (Figure 3A). Moreover, ethanol exposure led to a pronounced accumulation of inflammatory CD4^+^ T-cell subsets producing IFN-γ and IL-17 (Figure 3B,C).

**Figure 4:**
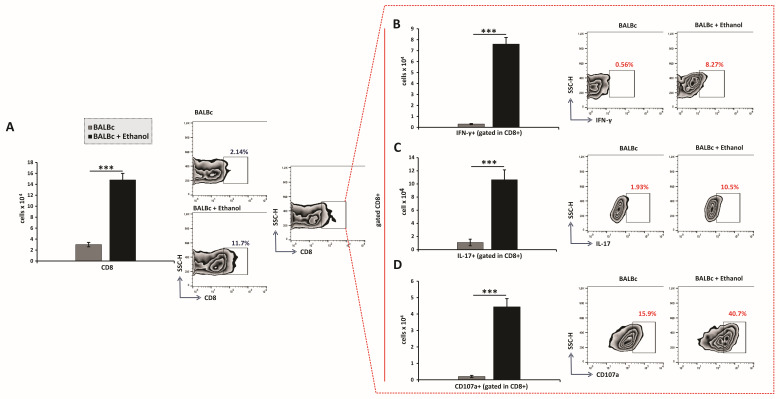
Ethanol exposure increases recruitment and effector polarization of CD8^+^ T lymphocytes: (**A**–**D**) Total numbers, subsets, and representative flow cytometry dot plots of CD8^+^ T cells. (**A**) Total CD8^+^ T-cell count. Quantification of CD8^+^ subsets producing (**B**) IFN-γ, (**C**) IL-17, and expressing (**D**) CD107a. Data are presented as mean ± SEM; *n* = 10 mice per group. * *p* < 0.05, ** *p* < 0.01, *** *p* < 0.001.

Further flow cytometric analysis revealed that ethanol treatment also increased the total number of gastric CD8^+^ T lymphocytes (Figure 4A), including IFN-γ- and IL-17-producing subsets (Figure 4B,C). Notably, a significantly higher frequency of activated CD107a^+^ CD8^+^ T cells was detected in the lamina propria of ethanol-treated animals compared with control mice (Figure 4D). Therefore, these findings demonstrate that ethanol exposure drives the expansion and functional activation of both CD4^+^ and CD8^+^ T-cell subsets, thereby amplifying adaptive immune responses that contribute to acute gastric mucosal injury.

## 4. Discussion

In this study, we showed that ethanol-induced gastric injury is accompanied not only by severe epithelial damage, a robust inflammatory response, and epithelial cell death but also by substantial recruitment and activation of CD4^+^ and CD8^+^ T lymphocytes. Additionally, dendritic cells, via IL-12, may serve as a crucial link between epithelial damage and the activation of these cells. These findings extend the current paradigm of gastric ulcer pathogenesis, which has traditionally emphasized innate immune mechanisms, by demonstrating that cellular immunity may also contribute significantly to acute gastric injury.

The ethanol-induced gastric injury model is a well-established and widely used experimental approach for investigating the pathogenesis of gastric ulcers and evaluating the efficacy of anti-ulcer therapies. Its short experimental duration, typically 1–3 h, makes it highly reproducible and suitable for capturing the early phase of mucosal damage and inflammatory activation. Moreover, ethanol is one of the most common etiological factors contributing to gastric damage in humans [14,18,21]. Ethanol-induced gastric mucosal injury arises through the combined effects of oxidative stress, inflammation, and epithelial cell death. Ethanol disrupts the oxidant–antioxidant balance, leading to increased reactive oxygen species (ROS), elevated lipid peroxidation, and reduced activity of antioxidant enzymes such as superoxide dismutase and catalase, thereby compromising epithelial barrier integrity [18]. In parallel, ethanol promotes the release of pro-inflammatory cytokines (IL-1β, TNF-α, and IFN-γ) and chemokines such as IL-8 and CXCL4, which recruit neutrophils to the mucosa and amplify tissue damage through myeloperoxidase release, ROS production, and proteolytic activity [14]. Ethanol also activates NF-κB and MAPK pathways, while NLRP3 inflammasome activation contributes to caspase-1-dependent IL-1β maturation, reinforcing the inflammatory response [18,21]. This pro-inflammatory environment promotes multiple cell death pathways, including necrosis, apoptosis, and dysregulated autophagy, which may fail to maintain epithelial integrity and further contribute to mucosal cell loss [18,21]. Altogether, these disruptions erode the protective mucosal barrier and culminate in hemorrhagic injury, pronounced edema, fragile tissue architecture, and the development of acute ulcerative lesions [14,18,21]. Consistent with these mechanisms, our macroscopic and histological analyses revealed extensive mucosal disruption in ethanol-treated mice (Figure 1A–E). The accompanying local increases in pro-inflammatory cytokines such as TNF-α, IL-1β, IL-17, and IFN-γ (Figure 1F) indicate a profound pro-inflammatory milieu driving gastric tissue destruction, further confirming the successful establishment of a gastric injury model. In parallel, the concomitant rise in IL-10 highlights an active counter-regulatory response, suggesting an attempt to limit the extent of mucosal injury.

Although the contribution of innate immune cells such as neutrophils and macrophages to sterile acute gastric injury is previously established [3,4], the involvement of adaptive immunity in this setting remains poorly defined. By contrast, in *H. pylori* gastritis, T-helper lymphocytes are recognized as central orchestrators of pro-inflammatory responses and key determinants of disease outcome. Early infection is characterized by a strong Th1 bias, with IFN-γ produced by Th1 cells activating macrophages and enhancing their phagocytic and bactericidal activity against *H. pylori* [23]. However, sustained Th1 activity promotes chronic inflammation that predisposes to gastritis and peptic ulcer formation [24]. Emerging evidence also indicates that Th17 cell responses may precede Th1 responses during the initial phase of *H. pylori* infection [25]. Th17 cells predominantly produce IL-17, which in turn induces chemokine expression and drives neutrophil recruitment [26]. Consistent with this, our results showed that ethanol administration led to an increased accumulation of CD4^+^ cells, including a greater number of IFN-γ- and IL-17-producing subsets (Figure 3A–C). In parallel, ethanol administration further increased the accumulation of CD8^+^ cells, including an increase in the number of CD107a^+^-expressing and IFN-γ- and IL-17-producing CD8^+^ subsets, indicating marked degranulation and activation (Figure 4A–D). These observations highlight an unrecognized role for CD8^+^ T cells in sterile acute gastric inflammation. Although CD8^+^ T cells are key defenders of mucosal tissues [27], their functions in the gastric inflammation, even during *H. pylori* infection remains poorly characterized. A recent study by Koch et al. [28] demonstrated that the first T cells to infiltrate the stomach during *H. pylori* infection are cytotoxin-associated gene A (CagA)-specific tissue-resident memory CD8^+^ T cells, which dominate the gastric T cell–driven immune response during the early chronic phase of *H. pylori* infection. In models of sterile tissue injury, such as cisplatin-induced kidney damage and concanavalin A-induced hepatitis, CD4^+^ T cells producing IFN-γ and IL-17, as well as CD8^+^ T cells producing IFN-γ, have been shown to contribute to tissue damage [29,30]. Regarding the role of IL-17–producing CD8^+^ T cells, they share phenotypic and cytokine characteristics with Th17 cells and have been implicated in the pathogenesis of multiple inflammatory diseases [31].

DCs, professional antigen-presenting cells activated by pathogen- and damage-associated molecular patterns (PAMPs and DAMPs) [32], have been consistently detected in inflamed gastric tissue in both humans and mice, although their presence in healthy gastric mucosa remains debated [33,34]. Consistent with this, in our study, ethanol-treated mice demonstrated a pronounced accumulation of DCs, accompanied by upregulated expression of the costimulatory molecule CD86 and enhanced IL-12 production (Figure 2A–C), indicative of a functionally activated state [35,36]. IL-12 is a central cytokine secreted by DCs that orchestrates Th1 polarization and drives IFN-γ production in CD4^+^ T cells [36]. This axis is well documented in *H. pylori*-induced gastric inflammation, where DCs-derived IL-12 promotes Th1-driven inflammation [34,37]. Moreover, it is well established that DCs, via CD86 expression and IL-12 secretion, deliver the second and third signals essential for the full activation of CD8^+^ T cells [38]. Extending these paradigms, our data suggest that ethanol-induced gastric injury engages a similar mechanism, whereby DCs activation promotes effector T-cell responses that amplify mucosal inflammation, supporting the concept of DCs as an interface linking innate and adaptive immunity.

**Figure 5:**
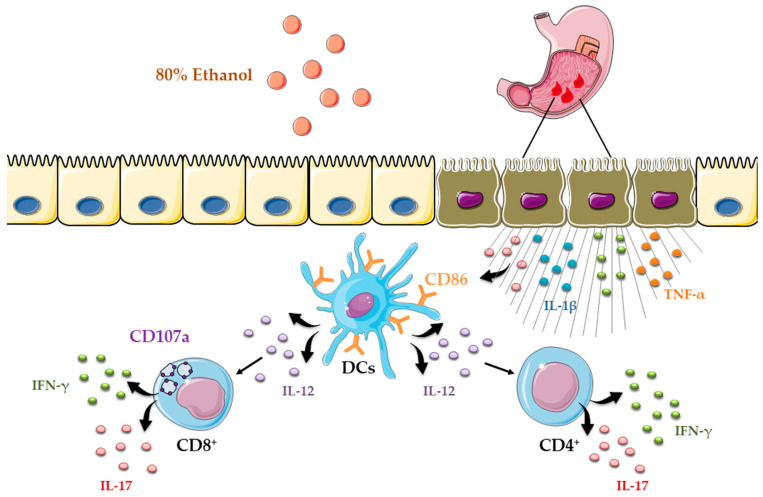
Schematic overview of dendritic cell-driven T helper and cytotoxic T-cell responses in ethanol-induced gastric injury.

Importantly, ethanol-induced gastric injury is not only characterized by inflammation but also by epithelial apoptosis [21]. We showed robust upregulation of the pro-apoptotic protein Bax and activation of caspase-3 in the gastric mucosa following ethanol exposure (Figure 1G), consistent with apoptotic cell death. Based on these findings, epithelial apoptosis in ethanol-induced gastric injury may arise from several mechanisms, including intrinsic pathways triggered by DNA damage, oxidative stress, and hypoxia, extrinsic pathways driven by pro-inflammatory cytokines such as TNF-α, and additional contributions from CD8^+^ T-cell cytotoxic activity [39].Ethanol exposure causes severe gastric epithelial damage and triggers the release of pro-inflammatory cytokines alongside DAMPs, which together activate DCs. Activated DCs upregulate the costimulatory molecule CD86 and secrete IL-12, thereby driving the recruitment and polarization of CD4^+^ T cells producing IFN-γ and IL-17. In parallel, ethanol promotes the accumulation of CD8^+^ T cells that also secrete IFN-γ and IL-17 and display the degranulation marker CD107a, indicative of cytotoxic activity (Figure 5). Collectively, these innate and adaptive immune responses intensify epithelial injury and engage regulated cell death pathways, shaping the extent of acute gastric mucosal damage. Several limitations should be considered when interpreting these findings. The work was performed in an acute murine model at a single time point, which limits insight into the temporal evolution of dendritic cell and T-cell responses. Moreover, the pathogenic contribution of acquired immune cells was inferred from correlative rather than functional evidence, and human validation is still lacking. Future research should address these gaps by employing longitudinal and functional approaches in gastric mucosal injury.

## 5. Conclusions

In conclusion, our study demonstrates that ethanol exposure induces gastric mucosal damage and triggers a robust pro-inflammatory cascade characterized by the pronounced expansion and activation of dendritic cells, increased IL-12 expression, and the expansion of CD4^+^ and CD8^+^ T cells, which exhibit strong IFN-γ and IL-17 secretion along with marked degranulation. Together, these immune responses exacerbate epithelial damage and activate regulated cell death mechanisms, ultimately determining the severity of acute gastric mucosal injury. These findings refine the prevailing view that ethanol-induced acute gastric injury is driven predominantly by innate pathways by demonstrating that cellular adaptive immunity is mobilized early and contributes to the amplification of tissue damage. Overall, these findings provide initial insights into the contribution of T helper and cytotoxic T cells to acute sterile gastric injury and pave the way for further clarification of regulatory mechanisms and the development of novel immunomodulatory strategies to mitigate epithelial damage in gastric disease.

## Data Availability

The original contributions presented in this study are included in the article. Further inquiries can be directed to the corresponding author.

## Abbreviations

NSAID	Nonsteroidal anti-inflammatory drug
CD	Cluster of differentiation
PBS	Phosphate-buffered saline
HAI	Histological activity index
ELISA	Enzyme-linked immunosorbent assay
TNF-α	Tumor necrosis factor alpha
IL-1β	Interleukin 1 beta
IL-17	Interleukin 17
IFN-γ	Interferon gamma
IL-10	Interleukin 10
RNA	Ribonucleic acid
cDNA	Complementary deoxyribonucleic acid
PCR	Polymerase chain reaction
Ct	Cycle threshold
FBS	Fetal bovine serum
HBSS	Hank’s balanced salt solution
EDTA	Ethylenediaminetetraacetic acid
DTT	Dithiothreitol
FITC	Fluorescein isothiocyanate
PE	Phycoerythrin
PerCP	Peridinin–chlorophyll protein
APC	Allophycocyanin
SEM	Standard error of the mean
DC	Dendritic cell
Th1	T helper 1 cell
CagA	Cytotoxin-associated gene A
PAMPs	Pathogen-associated molecular patterns
DAMPs	Damage-associated molecular patterns
ROS	Reactive oxygen species
NLRP-3	NOD-like receptor family pyrin domain-containing 3
